# Antidepressent Effect of Two New Benzyl Derivatives from Wild Strawberry *Fragaria vesca* var. *nubicola* Lindl. ex Hook.f.

**DOI:** 10.3389/fphar.2017.00469

**Published:** 2017-07-25

**Authors:** Sadia Naz, Umar Farooq, Ajmal Khan, Haroon Khan, Nasiara Karim, Rizwana Sarwar, Javid Hussain, Abdur Rauf

**Affiliations:** ^1^Department of Chemistry, COMSATS Institute of Information Technology Abbottabad, Pakistan; ^2^UoN Chair of Oman Medicinal Plants and Marine Products, University of Nizwa Nizwa, Oman; ^3^Department of Pharmacy, Abdul Wali Khan University Mardan, Pakistan; ^4^Department of Pharmacy, University of Malakand Chakdara, Pakistan; ^5^Department of Biological Sciences and Chemistry, College of Arts and Sciences, University of Nizwa Nizwa, Oman; ^6^Department of Chemistry, University of Swabi Ambar, Pakistan

**Keywords:** benzyl derivatives, *Fragaria vesca* var. *nubicola*, Rosaceae, antidepressant activity, anti-immobility

## Abstract

Two new benzyl derivatives were isolated from ethyl acetate fraction of wild strawberry, *Fragaria vesca* var. *nubicola* Lindl. ex Hook.f. The structures of these compounds were elucidated to be 5-(4-hydroxy-3-methoxyphenethyl)-7-methoxy-2H-chromen-3-ol (**1**) and 5-(4-hydroxy-3-methoxyphenethyl)-4,7-dimethoxy-2H-chromen-3-ol (**2**) based on spectroscopic data through IR, UV, ^1^H-NMR, ^13^C-NMR along with two dimensional (2D) techniques HMBC, HMQC, and COSY. Both compounds **1** and **2** were studied in tail suspension and forced swim tests for antidepressant like effects. A significant dose dependent antidepressant like effect was observed by causing spontaneous anti-immobility at various test doses upon intraperitoneal administration.

## Introduction

*Fragaria vesca* var. *nubicola* Lindl. ex Hook.f.; commonly known as wild strawberry is synonym of *Fragaria nubicola* (Lindl. ex Hook.f.) Laciata. It is a perennial herb belonging to family Rosaceae that mostly grows along roadsides and in forests. The family Rosaceae comprises of 85 genera and nearly about 3000 species widely distributed in Europe and North America while represented by 27 genera and about 160 species in Pakistan (Stewart, 1972; Mabberley, 2008).

The vegetative parts of *F. vesca* var. *nubicola* has been used as stimulant, diuretic agent, detoxifying agent and for treatment of diarrhea (Neves et al., 2009). The achenes and thalamus parts of *F. vesca* has been reported to have high phenolic content and showed good antioxidant activity (Cheel et al., 2007). Literature revealed that fruits as well as whole plant of *F. vesca* var. *nubicola* has potential analgesic and anti-oxidant activity (Kanodia and Das, 2008; Kanodia et al., 2011). Similarly, the ethanolic extract of *F. vesca* var. *nubicola* possesses anti-convulsant activity and is effective for treatment of epilepsy (Patil et al., 2012).

Strawberry fruits reported to have phenolic compounds like ellagic acid, ellagic acid-glycoside, coumaryl glycoside along with various anthocyanidin as their glycosides. The fruits are also reported to have anti-oxidant, anticancer, anti-inflammatory and anti-neurodegenerative properties (Hannum, 2004; Seeram et al., 2006). As previously reported the phytochemical investigation showed agrimoniin; an anti-tumor and antidiarrheal agent as major ellagitannins in *F. vesca* (Miyamoto et al., 1987; Vrhovsek et al., 2012). The roots of *F. vesca* var. *nubicola* are rich in proanthocyanidin (Vennat et al., 1986). Depression is one of serious disease prevailing all over the world affecting 13–20% population (Licinio and Wong, 1999). The discovery of new antidepressants is of utmost importance as significant proportion of patients develops resistance against medicines already available in market. Plants could be an effective approach for discovery of new antidepressant agents that can act via different mechanisms (Zhang, 2004).

Owing to its multiple traditional uses and strong phytochemical background of *F. vesca* var. *nubicola*, the current study was designed for isolation and characterization of bioactive secondary metabolites followed by their evaluation for antidepressant potential in various animal models.

## Materials and Methods

### Experimental Procedure

The EI-MS and HR-EI-MS analysis were done using double focusing Varian MAT-312 spectrometer and Bruker AMX-500 MHz spectrometer was used for ^1^H-NMR and ^13^C-NMR spectra. The chemical shift values were reported in ppm and scalar coupling in Hertz (Hz) using tetramethyl silane (TMS) as internal standard. The TLC analysis was done by using pre coated silica gel plates while columns were packed using E. Merck 230–400 mesh and 70–230 mesh and UV active compounds were detected by using ceric sulfate in 10% H_2_SO_4_ solution.

The IR and UV spectra were recorded through Hitachi JASCO-320-A and Hitachi UV-3200 spectrophotometer respectively.

### Plant Material

The whole plant of *F. vesca* var. *nubicola* (6 kg) was collected from Hazara division of Khyber Pakhtunkhwa, Pakistan in May 2015 and a voucher specimen (no 8473) has been deposited in herbarium Department of Botany Postgraduate College, Abbottabad Pakistan.

### Extraction and Isolation

**FIGURE 1:**
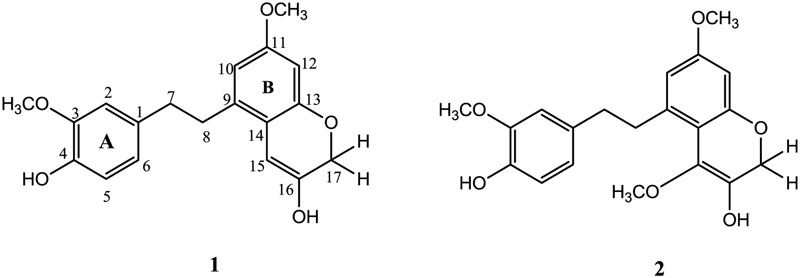
Structures of compounds **1** and **2** (Zhan et al., 2016).

The whole shade dried plant was ground into fine powder and extracted with methanol at room temperature and filtered thrice. The vacuum rotary evaporator was used to get crude extract from filtrate. The crude extract (450 g) was partitioned into four fractions as *n*-hexane (120 g), chloroform (75 g), ethyl acetate (150 g), and *n*-butanol (65 g). The ethyl acetate fraction was selected on the basis of TLC analysis and subjected to column chromatography by using *n*-hexane as gradient of ethyl acetate to 100% followed by methanol. Total 12 sub-fractions (A–L) were obtained and depending on TLC analysis four sub-fractions (G–J) were re-subjected to column chromatography to obtain compound **1** (12 mg) at *n*-hexane: ethyl acetate (40:60). Similarly compound **2** (7.8 mg) was obtained from re-column chromatography of sub-fractions (D–F) at polarity of *n*-hexane: ethyl acetate (45:55) (**Figure 1**).

### Characterization of Compound **1**

A colorless oil; UV (MeOH) λ_max_ 222 (2.9), 252 (3.6), 298 (3.9), 324 (4.6) nm; IR (KBr) υ_max_ 3372, 2920, 1640, 1610, 1230, 1019 cm^-1^; EI-MS m/z: 328 [M]^+^ (100), 310 (80), 296 (60), 292 (62), 264 (50), 260 (39), 228 (71), 150 (30) and 120 (45); HR-EI-MS: m/z [M^+^] Calcd. 328.1311 for Mol. formula C_19_H_20_O_5_; Observed 328.1305; ^1^H NMR (500 MHz, CDCl_3_) δ (ppm): 6.68 (1H, d = 2.4 Hz, H-2), 6.89 (1H, d = 8.1 Hz, H-5), 6.72 (1H, dd = 8.1, 2.4 Hz, H-6), 2.76 (2H, m, H-7), 2.94 (2H, m, H-8), 6.54 (1H, d = 2.6 Hz, H-10), 6.40 (1H, d = 2.6 Hz, H-12), 6.30 (1H, s, H-15), 4.28 (1H, d = 15.6 Hz, H-17), 3.80 (1H, d = 15.6 Hz, H-17), 3.74 (3H, s, 3-OCH_3_), 3.65 (3H, s, 11-OCH_3_); ^13^C NMR (125 MHz, CDCl_3_) δ (ppm): 137.2 (C-1), 119.2 (C-2), 149.9 (C-3), 148.3 (C-4), 115.7 (C-5), 124.8 (C-6), 40.2 (C-7), 38.9 (C-8), 145.8 (C-9), 107.9 (C-10), 162.1 (C-11), 102.3 (C-12), 160.6 (C-13), 109.7 (C-14), 104.4 (C-15), 150.6 (C-16), 64.3 (C-17), 58.4 (3-OCH_3_), 56.4 (11-OCH_3_).

### Characterization of Compound **2**

A colorless oil; UV (MeOH) λ_max_ 212 (3.6), 240 (2.8), 296 (4.1), 310 (3.9) nm; IR (KBr) υ_max_ 3380, 2960, 1650, 1230, 1102 cm^-1^; EI-MS m/z: 358 [M]^+^ (100), 340 (90), 326 (75), 322 (62), 294 (50), 290 (60), 264 (49), 258 (70), 226 (39), 148 (35), and 118 (43); HR-EI-MS: m/z [M^+^] Calcd. 358.1416 for Mol. formula C_20_H_22_O_6_; Observed 358.1409; ^1^H NMR (500 MHz, CDCl_3_) δ (ppm): 6.60 (1H, d = 2.1 Hz, H-2), 6.90 (1H, d = 8.4 Hz, H-5), 6.76 (1H, dd = 8.4, 2.1 Hz, H-6), 2.62 (2H, m, H-7), 2.82 (2H, m, H-8), 6.50 (1H, d = 2.3 Hz, H-10), 6.45 (1H, d = 2.3 Hz, H-12), 4.20 (1H, d = 16.1 Hz, H-17), 3.81 (1H, d = 16.1 Hz, H-17), 3.74 (3H, s, 3-OCH_3_), 3.59 (3H, s, 11-OCH_3_), 3.57 (3H, s, 15-OCH_3_); ^13^C NMR (125 MHz, CDCl_3_) δ (ppm): 138.4 (C-1), 115.4 (C-2), 150.1 (C-3), 144.6 (C-4), 117.7 (C-5), 123.8 (C-6), 37.4 (C-7), 36.8 (C-8), 146.3 (C-9), 111.6 (C-10), 162.4 (C-11), 99.6 (C-12), 159.3 (C-13), 107.8 (C-14), 116.4 (C-15), 136.6 (C-16), 60.9 (C-17), 57.1 (3-OCH_3_), 55.1 (11-OCH_3_), 56.9 (15-OCH_3_).

### Acute Toxicity Study of Compounds 1 and 2

The acute toxicity of compounds **1** and **2** was determined by using swiss albino mice, according to the method described by Irwin (Irwin, 1968). The animals were divided into six groups (*n* = 9). One group served as a control and received vehicle orally. Briefly, compounds **1** and **2** each were administered at the dose level of 50,100, 200, and 300 mg/kg to each mouse orally. Each animal was subjected to various parameters including writhing, convulsions, aggressiveness, hypersensitivity, salivation, lacrimation, spontaneous activity, ataxia, and catalepsy 30 min prior to injection (baseline) and then at 0 (straight after injection), 30 and 60 min, 24, 48, and 72 h and 1 week after administration for any kind of behavioral, physical, and pharmacological toxic effects.

### Tail Suspension Test

Mice were hung by their tail on the tail hanger using sticky tape for tail fixation, at approximately 1 cm from the end of the tail. The hanger was fixed in the black plastic box (20 cm × 20 cm × 45 cm) with the opening at the top front. The distance between the hanger and floor was approximately 40 cm. The mouse was suspended in the air by its tail and the immobility time was recorded over a period of 5 min. The duration of immobility was defined as the absence of all movements except for those required for respiration (Steru et al., 1985).

### Forced Swim Test

For forced swim test in mice, the method reported by Porsolt et al. (1978) was adopted. Swim sessions of animal were performed in individual glass cylinders (46 cm tall × 20 cm diameter) containing 30 cm of water at 24 ± 1°C. Following both swim sessions, mice were removed from the cylinders, dried with paper towels, and placed in heated cages for 15 min and then returned to their home cages. Various doses of test compounds **1** and **2** like 10, 30, and 100 mg/kg i.p. and standard Imipramine (60 mg/kg) were administered during two swim sessions: an initial 15-min pretest followed 14 days later by a 5-min test.

### Research Ethics Committee Approval

It is certified that the Departmental Research Ethics Committee (DREC) reviewed the National Research Program for Universities (NRPU) research grants application of the project entitled “Anxiolytic and Antidepressant Activities of Selected Natural product (Glycosides and Flavonoids).” The principal investigator of the project is Dr. Nasiara Karim, Assistant Professor, Department of Pharmacy, University of Malakand.

The Committee approves (DAEC/Pharm/2017/01) the study to be conducted in the present form, and expects to be inform about any revision in the protocol and subject/patient information/informed consent (where applicable).

### Statistical Analysis

Data are presented as mean ± SEM of six mice. A one-way factorial analysis of variance (ANOVA) followed by *post hoc* Dunnett’s test for multiple comparisons was used to assess behavior in tests. The *P*-value < 0.05 was considered as significant.

## Results

Two new benzyl derivatives have been isolated from ethyl acetate fraction of *F. vesca* var. *nubicola* through successive column chromatography, while characterization was made through various spectroscopic techniques and comparison with literature. The isolated compounds **1** and **2** exhibited significant antidepressant like effects in tail suspension and forced swim tests.

Compound **1** was isolated as colorless oil with molecular formula C_19_H_20_O_5_ as suggested by HR-EI-MS with highest molecular ion peak at m/z 328.1305 [M^+^] and m/z values obtained were 310, 296, 292, 264, 260, 228, 150, and 120. The UV spectrum showed absorption band at λ_max_ 222 (2.9), 252 (3.6), 298 (3.9), 324 (4.6) and presence of various groups like hydroxyl group, alkane, alkene, aromatic moiety, and C-O were suggested by absorption bands in IR spectrum at υ_max_ 3372, 2920, 1640, 1610, and 1230, respectively.

The ^1^H-NMR spectrum revealed the presence of five aromatic protons at chemical shift values of δ_H_ 6.54 (1H, d = 2.6 Hz, H-10), 6.40 (1H, d = 2.6 Hz, H-12), 6.68 (1H, d = 2.4 Hz, H-2), 6.89 (1H, d = 8.1 Hz, H-5), and 6.72 (1H, dd = 8.1, 2.4 Hz, H-6). Similarly two methylene proton signals appeared at δ_H_ 2.76 (2H, m, H-7) and 2.94 (2H, m, H-8), while a methine proton resonated at δ_H_ 6.30 (1H, s, H-15) along with two oxidized protons at δ_H_ 4.28 and 3.80 showing germinal coupling with each other (1H, d = 15.6 Hz each) as given in **Table 1**. The ^1^H-NMR spectrum also suggested the presence of two methoxy groups at δ_H_ 3.74 (3H, s, -OCH_3_) and δ_H_ 3.65 (3H, s, OCH_3_).

**Table 1:** ^1^H NMR (500 MHz, CDCl_3_), δ_H_ in ppm and ^13^C NMR (125MHz, CDCl_3_), δ_C_ in ppm of compounds **1** and **2.**

Position	Compound 1		Compound 2	
	^1^H-NMR	^13^C-NMR	^1^H-NMR	^13^C-NMR
	(δ_H_ ppm) and multiplicity	(δ_C_ ppm)	(δ_H_ ppm)	(δ_C_ ppm)
1	–	137.2	–	138.4
2	6.68 (1H, d = 2.4 Hz)	119.2	6.60 (1H, d = 2.1 Hz)	115.4
3	–	149.9	–	150.1
4	–	148.3	–	144.6
5	6.89 (1H, d = 8.1 Hz)	115.7	6.90 (1H, d = 8.4 Hz)	117.7
6	6.72 (1H, dd = 8.1, 2.4 Hz)	124.8	6.76 (1H, dd = 8.4, 2.1 Hz)	123.8
7	2.76 (2H, m)	40.2	2.62 (2H, m)	37.4
8	2.94 (2H, m)	38.9	2.82 (2H, m)	36.8
9	–	145.8	–	146.3
10	6.54 (1H, d = 2.6 Hz)	107.9	6.50 (1H, d = 2.3 Hz)	111.6
11	–	162.1	–	162.4
12	6.40 (1H, d = 2.6 Hz)	102.3	6.45 (1H, d = 2.3 Hz)	99.6
13	–	160.6	–	159.3
14	–	109.7	–	107.8
15	6.30 (1H, s)	104.4	–	116.4
16	–	150.6	–	136.6
17	4.28 (1H, d = 15.6 Hz)	64.3	4.20 (1H, d = 16.1 Hz)	60.9
	3.80 (1H, d = 15.6 Hz)		3.81 (1H, d = 16.1 Hz)	
3-OCH_3_	3.74 (3H, s)	58.4	3.74 (3H, s)	57.1
11-OCH_3_	3.65 (3H, s)	56.4	3.59 (3H, s)	55.1
15-OCH_3_	–	–	3.57 (3H, s)	56.9

The ^13^C-NMR and DEPT spectrum revealed the presence of nineteen 19 carbon atoms including two methyl, three methylene, six methine, and eight quaternary carbon atoms. Two methoxy carbons resonated at δ_C_ 56.4 (C-11) and 58.4 (C-3) while three methylene carbons were observed at δ_C_ 40.2 (C-7), 38.9 (C-8), and 64.3 (C-17). ^13^C-NMR showed five aromatic methine carbon atoms at δ_C_ 102.3 (C-12), 107.9 (C-10), 119.2 (C-2), 115.7 (C-5), and 124.8 (C-6) with another methine carbon at δ_C_ 104.4 (C-15) as given in **Table 1**. The quaternary carbons resonated at δ_C_ 149.9 (C-3), 148.3 (C-4), 137.2 (C-1), 145.8 (C-9), 162.1 (C-11), 160.6 (C-13), 109.7 (C-14), and 150.6 (C-16).

**FIGURE 2:**
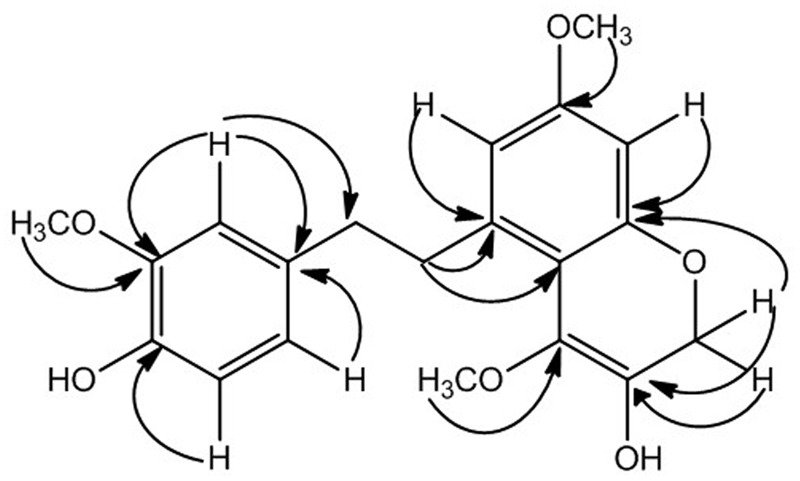
Important HMBC (→) correlations.

The data of HMBC spectrum was quite supportive for accurate placement of substituents in compound **1**. The HMBC correlation of methoxy protons at δ_H_ 3.74 showed strong correlation with C-3, C-4, and C-2 confirming its position at C-3 similarly second methoxy group (δ_H_ 3.65) was placed at position C-11 showing HMBC correlation with C-10, C-11, and C-12. The placement of two hydroxyl groups at C-4 and C-16 were established from HMBC correlations, like H-2 (δ_H_ 6.68) and H-6 (δ_H_ 6.72) showed connectivity with C-4 and correlation of H-17 and H-15 with C-16 respectively. The methylene protons at position C-7 showed strong HMBC correlations with C-1, C-2 and C-6, while protons at position C-8 showed HMBC correlations with C-9 and C-10 suggesting presence of bibenzyl group in compound **1** as depicted in **Figure 2** (Zhan et al., 2016). The HMBC correlations of methylene protons at C-17 showed strong correlation with C-13, C-15, and C-16 while correlation of H-15 with C-13, C-14, C-16, and C-17 formed an oxygen heterocyclic ring as shown in **Figure 1**. The structure of compound **1** was suggested as 5-(4-hydroxy-3-methoxyphenethyl)-7-methoxy-2H-chromen-3-ol on the basis of all spectral data and literature comparison (Zhan et al., 2016).

Compound **2** was isolated as colorless oil from ethyl acetate fraction of *F. vesca* var. *nubicola*. Its molecular formula was C_20_H_22_O_6_ as suggested by highest molecular ion peak at m/z 358.1409 [M^+^] while other m/z values were 340, 326, 322, 294, 290, 264, 258, 226, 148, and 118. The absorption bands in UV spectrum were obtained at λ_max_ 212 (3.6), 240 (2.8), 296 (4.1), 310 (3.9) and IR spectrum showed absorption bands at υ_max_ 3380, 2960, 1650, 1610, and 1230 suggesting presence of hydroxyl group, alkane, alkene, aromatic moiety, and C-O respectively.

The ^1^H-NMR and ^13^C-NMR data of compound **2** is quite identical to compound **1**. Compound **2** had one additional methoxy group which was evident from all spectral data. The ^1^H-NMR showed chemical shift value for three methoxy groups at δ_H_ 3.74 (3H, s, δ_C_ = 57.1) and 3.59 (3H, s, δ_C_ = 55.1) at position C-3 and C-11 similar to compound **1** with additional methoxy group at position C-15 having chemical shift value of δ_H_ 3.57 (3H, s, δ_C_ = 56.9). The ^1^H-NMR spectrum showed presence of five aromatic protons at δ_H_ 6.60 (1H, d = 2.1 Hz, H-2, δ_C_ = 115.4), 6.90 (1H, d = 8.4 Hz, H-5, δ_C_ = 117.7), 6.76 (1H, dd = 8.4, 2.1 Hz, H-6, δ_C_ = 123.8) for ring A along with δ_H_ 6.50 (1H, d = 2.3 Hz, H-10, δ_C_ = 111.6) having meta coupling with proton at δ_H_ 6.45 (1H, d = 2.3 Hz, H-12, δ_C_ = 99.6) at ring B.

^13^C-NMR spectrum and DEPT showed presence of twenty carbon atoms including three methoxyl group, three methylene, five methine, and nine quaternary carbons given in **Table 1**. The HMBC correlations of substituents were quite identical to those for compound **1**, the only difference observed was a methoxy group at position C-15 showing HMBC connectivity of protons with C-15, C-16 and C-14. On the basis of all spectral data and literature comparison the structure established for compound **2** was 5-(4-hydroxy-3-methoxyphenethyl)-4,7-dimethoxy-2H-chromen-3-ol.

### Acute Toxicity Tests

Acute toxicity studies showed that the administration of compounds **1** and **2** at the dose level of 50–300 mg/kg did not produce any significant change in the behavior of the animals as observed by lack of convulsions, respiratory distress, writhing, changes to reflex activity, or mortality. A slight sedation was observed at 300 mg/kg, however, the animals remained alert. At 24 h to 1 week, all animals seemed well with no observable changes in behavior or appearance. No deaths were observed up to 1 week of study.

### Effect in Tail Suspension Test

The effects of both compounds **1** and **2** in tail suspension tested are shown in **Table 2**. Both compounds **1** and **2** exhibited marked dose dependent antidepressant effects by reducing immobility time.

**Table 2:** Effect of compounds on immobility time (sec) of mice using tail suspension test.

Treatment	Dose (mg/kg)	Immobility time (sec)
Control	–	175.5 ± 10.5
Compound 1	10	170.4 ± 11.5
	30	140.5 ± 5.3^∗^
	100	110.35 ± 8.2^∗∗^
Compound 2	10	168.4 ± 13.5
	30	130.3 ± 7.5^∗^
	100	105.5 ± 10.3^∗∗^
Imipramine	60	60.5 ± 13.6^∗∗∗^

*All values are expressed in mean ± SEM (*n* = 6). ^∗^*P* < 0.05, ^∗∗^*P* < 0.01, ^∗∗∗^*P* < 0.001 compared to the vehicle group. Difference between groups were analyzed by analysis of variance (one-way ANOVA followed by Dunnett’s test).*

### Effect in Forced Swim Test

Forced swim test is another useful and commonly used assay for the determination of antidepressant like effects of compounds (Mohseni et al., 2017; Sakhaee et al., 2017). Both compounds **1** and **2** showed dose dependent antidepressant effect as given in **Table 3**. Both isolated compounds caused significant immobility time and thus showed their potential as antidepressant.

**Table 3:** Effect of compounds on immobility time (sec) of mice using forced swimming test.

Treatment	Dose (mg/kg)	Immobility time (sec)
Control	–	185.5 ± 12.5
Compound 1	10	178.5 ± 10.6
	30	115.5 ± 11.3^∗∗^
	100	83.5 ± 8.4^∗∗∗^
Compound 2	10	175.6 ± 13.5
	30	140.4 ± 7.8^∗^
	100	71.9 ± 10.5^∗∗∗^
Imipramine	60	76.5 ± 14.6^∗∗∗^

*All values are expressed in mean ± SEM (*n* = 6). ^∗^*P* < 0.05, ^∗∗^*P* < 0.01, ^∗∗∗^*P* < 0.001 compared to the vehicle group. Difference between groups were analyzed by analysis of variance (one-way ANOVA) followed by Dunnett’s test.*

## Discussion

The acute toxicity and behavioral test is mandatory for further detail studies. In our study both compounds were found safe with no behavior changes in the specified time, nor any mobility or mortality.

The TST and FST are reported to be the most commonly used predictive tests of antidepressant effects sensitive to the acute administration of antidepressant substances (Cryan et al., 2005). These behavioural tests are based on induction of immobility in rodents that face an inescapable situation and the antidepressant activity is indicated reduction in immobility time (Porsolt et al., 1977). In this study, compounds 1 and 2 displayed antidepressant like effects in both FST and TST. Although many antidepressant including monoamine oxidase inhibitors, tricyclics, selective serotonin reuptake inhibitors and selective noradrenaline reuptake inhibitors are available in the market for the treatment of various types of depressive disorders but even the first line antidepressant are facing many challenges in terms of unwanted effects (Kennedy, 2013; Johannessen et al., 2016).

The antidepressant like potential of natural isolated compounds is well-documented (Belozertseva et al., 2007; Pervez et al., 2016). Herbal therapies including the famous St. John’s wort have been found effective in the treatment of various depressive disorders. There has been renewed interest recently for novel pharmacotherapy from medicinal plants and compounds isolated from plant extracts. It is assumed that they might follow a different mechanistic pathway and better pharmacokinetic profile and thus better patient compliance. The compounds **1** and **2** may serve as leads for the development of newer antidepressant agents from natural sources. However, further detail studies are needed to ascertain their clinical status.

## Author Contributions

AK and UF conceived and designed the study. SN performed isolation and NK performed *in vivo* studies. JH analyzed the data. UF and AK wrote the manuscript with inputs and comments from all co-authors. All authors have read and approved the final version of the manuscript.

## Conflict of Interest Statement

The authors declare that the research was conducted in the absence of any commercial or financial relationships that could be construed as a potential conflict of interest.
